# From Wm. J. Cronyn, M. D.

**Published:** 1885-10

**Authors:** Wm. J. Cronyn

**Affiliations:** Dunkirk, N. Y.


### Communications to the Editor.

---

MESSRS. EDITORS—I noticed recently an article by Prof. Mezzeroff, entitled, the "Prevention and Cure of Cholera," in which he takes issue with Dr. Koch and other eminent writers and observers, as to the *primary* cause of the disease, claiming that it is due to absorption of a "poison gas," affecting the nerve centers, and not to animal or vegetable poison, or "microbes." While this question is being discussed by the theorists, practical men in the profession will busy themselves with thinking out proper means for the removal of *general causes* and remedies for the cure of this dread disease. The professor writes of cholera as it manifested itself in his own person, and then goes on to state the *treatment* and "*cure*," as directed by himself. He writes so confidently about his treatment being a positive cure for cholera, and his recommendations for its prevention so certain of success, that his article is worth analyzing for the sake of truth and benefit of humanity. For application over the stomach, he used capsicum, mustard, etc., and had his extremities rubbed with chloroform. Internally, he took brandy and chloroform (*two drams* of the latter), and, "fifteen minutes afterward," took the following: "one ounce sulphate of magnesia; two drams of chlorate of potash; one dram bi-carbonate of soda mixed in water." He states that he had a "terrible fight to retain this on the stomach for twenty minutes, when some of it was rejected." The wonder is that he retained any part of it. He attributes his recovery to this treatment, and claims the idea of compounding this mixture in cholera as *original*. Now, the facts are, that everything that was done in his case has been done scores of times for others; and with what result? Some lived, some died. If the professor imagines that he was the pioneer in this treatment, let him take the trouble to read the Cholera Reports of the Royal College of Physicians, London, and he will make up his mind that it is mighty difficult to suggest any treatment which has not been tried and found wanting in many cases. Dr. William Stevens, of Coldbath Fields, gave the above mixture as long ago as 1832. So this does away with the professor's claim for originality. But, if he had not taken the mixture, and had relied entirely on the brandy and chloroform, how does he know but that he would have recovered? I believe he would. The chloroform was absorbed, and acted as a powerful sedative to the "nerve centers, allaying the irritation and arresting the violent vomiting, purging and cramps, and gave a chance for re-action." Furthermore, will some chemist tell us what the above compound really was when it was in the stomach "twenty minutes"? In contact with the brandy and the vitiated secretions of the stomach, might not the compound be changed so that we would get *Sulphite Soda*, a *Carbonate* of Magnesia and *Chloride of Sodium?* Who knows? He seems to have great faith in the preventive qualities of chlorate of potash, pepper and epsom salts. He claims this as an original idea, but acknowledges that the capsicum part of the application suggested itself to his mind because he had seen it freely used in warm countries for cramps and diarrhœa. I have seen curry much used in warm countries, but I would never think of applying it as a poultice in cholera. This reminds me that the basis of curry is red and black pepper, and is much used as a kind of sauce in India, where it is said they have cholera all the year round, and thousands die from it. If the followers of Mohammet, who go on pilgrimages to Mecca, would only mix epsom salts and chlorate of potash in their curry, they would be saved—I mean from cholera, according to Professor Mezzeroff. He states that he had *proved that Sulphate of Magnesia was the best medicine to cure inflammation of the brain and nerve centers of the spine*" (queer, "nerve centers of the spine.")* Do we have this in cholera? How did it "cure;" by absorption and direct effect, or by derivative action? If disturbances of the brain and nerve

*Prof. John C. Dalton, of New York City, made examination after death of *thirty-three* cases of cholera, and reports *"the spinal cord offers nothing abnormal."*

centers be caused through reflex action by an irritant in the stomach or bowels, then sulphate of magnesia is good—just as good as any other cathartic. Right here, allow me to digress. It is to be hoped that this latter quotation from the professor's article is a *fact*, for it is a "pointer" to two or three medical bell-weathers in New York and Philadelphia, who are "clean cracked" on "*spinal irritation.*" Fifty years ago, when doctors didn't know what to call an illness, they said it was *splenic;* twenty-five years later, the poor *liver* had to take the curse of their ignorance, but for the last few years "*spinal irritation*" is the cause of all obscure pains and nervous symptoms. What is the cure? A dose of salts, according to Professor Mezzeroff; he has "proved it." The medical bell-weathers aforesaid will, therefore, please take notice, and not hereafter resort to moxa or white-hot iron for the cure of spinal irritation, which, I believe, may often be present *as a symptom*, but not once in a hundred cases as a disease, the opinions of these voluminous writers to the contrary notwithstanding. But to the cholera question, I quite agree with Dr. Mezzeroff as to importance of cleanliness of body, and supply of pure drinking water, but I don't like his restriction of diet, nor do I believe it at all essential. He tells us, if we would not have cholera, we "*must shun acid fruits, all cabbages, pickles, vinegar, peas, etc., etc.* What, abstain from these, and die of scurvy! Now, this is an original *cure* for cholera. He says one may "eat *corn* and *green beans*, but not peas, for peas produce diarrhœa." Now, as a physician, he must be aware of the fact that the eating of corn is a frequent cause of diarrhœa, and green beans will cause in many urticaria (nettle rash). He warns us not to drink water, unless it is boiled, but directs that we "drink good whisky or brandy, with plenty of black pepper, mixed with sugar and water." Not a bad dose to take, but an excellent thing, if continued for a season, to produce chronic inflammation of the stomach, fatty degeneration of the liver and atrophy of the absorbents, which would be very likely to land a poor devil helpless in the hands of a spinal irritationist. Thanks, professor, but I think I would rather have the cholera. I will not go into the sanitary and hygienic means for the prevention of cholera, further than to say that the profession is well agreed as to the sanitary and hygienic means that should be used. But they get "by the ears" at once when they come to the treatment, and all who have had experience with the disease, will acknowledge that there is no specific; that no matter what treatment is adopted, many will die. I believe, however, that in the first and second stages of the disease, much may be done to save life. After the stomach and bowels are evacuated, the violent vomiting, purging and cramps continue because of the congested condition of the capillaries of these and other internal organs; we have cold surfaces and extremities, because of this unequal circulation and sudden loss of the fluids of the body, consequently violent contractions of the voluntary muscles and spasm, also of the involuntary ones, and all these because of *some irritation of the nerve centers.* Well, what is to be done; no matter whether the *irritant* be a "poison gas" or some other thing? Why, *allay irritation of these nerve centers.* Stop their fury! How? By giving sedatives! Produce quiet, rest, sleep! A sixth to quarter grain of sulphate of morphia in solution, injected under the skin, and five or ten minutes afterwards give inhalation of chloroform; restore circulation and warmth to surfaces and extremities by swathing the body in *hot wet* flannels,covered with oiled silk, or oiled cotton, and keep hot by applying hot bricks, bottles of hot water, etc. The vomiting and purging ceases; the cramps are relieved. Your anodynes have deadened the diseased sensibility of the nerve centers. You have quieted their fury, cholera and death have passed, and the doctor triumphs. Give *nothing* by the stomach (it probably would not retain anything, unless it be a few mouthfuls of hot water,) for several hours, then carefully give concentrated nourishment. It should be borne in mind that the action of chloroform is greatly augmented when inhaled after the hypodermic injection of morphia, a few breaths being sufficient to produce sleep. It would be difficult to suggest a remedy or means of cure that has not been tried. Prof. Austin Flint mentions the fact of a writer in a *Medical Journal* recommending "hog's bristles, or hair from a cow's tail, burned to a cinder," as a cure for cholera, and this in all honesty. The treatment by sedatives and anæsthetics, as recommended above, will not avail after the period of collapse, when the mucous membrane of the stomach and bowels have become changed, and is sloughing off, together with softening of other tissues of internal organs. I advocate their use only in the first and second stages of the disease. Respectfully,

WM. J. CRONYN, M. D.

DUNKIRK, N. Y.

